# The WNK-SPAK/OSR1 Kinases and the Cation-Chloride Cotransporters as Therapeutic Targets for Neurological Diseases

**DOI:** 10.14336/AD.2018.0928

**Published:** 2019-06-01

**Authors:** Huachen Huang, Shanshan Song, Suneel Banerjee, Tong Jiang, Jinwei Zhang, Kristopher T. Kahle, Dandan Sun, Zhongling Zhang

**Affiliations:** ^1^ Department of Neurology, The First Affiliate Hospital, Harbin Medical University, Harbin, Heilongjiang, China.; ^2^Department of Neurology, University of Pittsburgh, Pittsburgh, PA, USA.; ^3^Institute of Biomedical and Clinical Sciences, University of Exeter Medical School, Hatherly Laboratory, Exeter, EX4 4PS, UK.; ^4^Departments of Neurosurgery, Pediatrics, and Cellular & Molecular Physiology, Centers for Mendelian Genomics, Yale School of Medicine, New Haven, CT, USA.; ^5^Veterans Affairs Pittsburgh Health Care System, Geriatric Research, Education and Clinical Center, Pittsburgh, PA, USA.

**Keywords:** brain edema, cell volume regulation, ischemic stroke, KCCs, NKCC1, WNK-SPAK/OSR1

## Abstract

In recent years, cation-chloride cotransporters (CCCs) have drawn attention in the medical neuroscience research. CCCs include the family of Na^+^-coupled Cl^-^ importers (NCC, NKCC1, and NKCC2), K^+^-coupled Cl^-^ exporters (KCCs), and possibly polyamine transporters (CCC9) and CCC interacting protein (CIP1). For decades, CCCs have been the targets of several commonly used diuretic drugs, including hydrochlorothiazide, furosemide, and bumetanide. Genetic mutations of NCC and NKCC2 cause congenital renal tubular disorders and lead to renal salt-losing hypotension, secondary hyperreninemia, and hypokalemic metabolic alkalosis. New studies reveal that CCCs along with their regulatory WNK (Kinase with no lysine (K)), and SPAK (Ste20-related proline-alanine-rich kinase)/OSR1(oxidative stress-responsive kinase-1) are essential for regulating cell volume and maintaining ionic homeostasis in the nervous system, especially roles of the WNK-SPAK-NKCC1 signaling pathway in ischemic brain injury and hypersecretion of cerebrospinal fluid in post-hemorrhagic hydrocephalus. In addition, disruption of Cl^-^ exporter KCC2 has an effect on synaptic inhibition, which may be involved in developing pain, epilepsy, and possibly some neuropsychiatric disorders. Interference with KCC3 leads to peripheral nervous system neuropathy as well as axon and nerve fiber swelling and psychosis. The WNK-SPAK/OSR1-CCCs complex emerges as therapeutic targets for multiple neurological diseases. This review will highlight these new findings.

Maintaining intracellular ionic homeostasis is essential for physiological function in all cells. A family of electroneutral transporters, the SLC12A family of CCCs (cation-chloride cotransporters), and their upstream regulatory serine-threonine kinases, the WNK (Kinase with no lysine (K)), SPAK (Ste20-related proline-alanine-rich kinase) as well as OSR1 (oxidative stress-responsive kinase-1), play integral roles in the regulation of intracellular Na^+^, K^+^, and Cl^-^ homeostasis as well as cell volume homeostasis [1-3]. The CCC family is comprised of the Na^+^, K^+^-coupled Cl^-^ importers (NCC, NKCC1, and NKCC2) and the K^+^-coupled Cl^-^ exporters (KCC1-4) [4, 5]. Isoforms of WNK are phosphorylated (activated) in response to osmotic stress or when intracellular Cl^-^ levels are low, and subsequently phosphorylate the related downstream kinases SPAK and/or OSR1 [6, 7]. Activated SPAK and/or OSR1 in turn stimulates NCC, NKCC1, NKCC2 via protein phosphorylation, but inhibits KCCs (with phosphorylation), via a reciprocal regulatory mechanism [8]. The inverse regulation of these ion cotransporters is driven by the same kinase-phosphatase signaling pathway to coordinate cellular Cl^-^ efflux and influx in the cells to maintain Cl^-^ homeostasis and avoid unnecessary ATP consumption. Generally, NKCC1, NKCC2, and NCC mediate Na^+^, K^+^, and Cl^-^ influx, while KCC1-4 mediate K^+^ and Cl^-^ efflux [9]. Abnormal function of the SLC12A family members was first associated with kidney dysfunction and diseases. Mutations of these CCCs can cause congenital renal tubular disorders such as renal salt-wasting syndromes [Gitelman syndrome (GS) and Bartter syndrome (BS)], and lead to secondary hyperaldosteronism and hypokalemic metabolic alkalosis (Table 1). Both GS and BS are caused by autosomal recessive gene mutations of NCC in the distal convoluted tubules (DCT) and NKCC2 in the thick ascending limb (TAL) of the loop of Henle (Table 1) [10]. BS is divided into 5 types, each with different causes. Type I BS is caused by a forementioned SLC12A mutation (NKCC2) [11]. Other causes of BS include mutations in the potassium voltage-gated channel subfamily J member 1 (KCNJ1) for Type II BS, chloride channel Kb (ClC-Kb) for Type III BS, Bartter syndrome, infantile, with sensorineural deafness (BSND) for Type IV BS, and calcium-sensing receptor (CaSR) for Type V BS [11]. All of these disorders are caused by the disruption of ion homeostasis [12, 13]. New studies suggest that the WNK-SPAK/OSR1-CCC pathway is also involved in neurological diseases such as ischemic stroke and neuropathy [14-19]. This review will give readers an update on the emerging roles of WNK-SPAK/OSR1-CCC signaling pathway in neurological diseases.

## WNK-SPAK/OSR1 kinase complex and Cl^-^ cotransporters in cell volume regulation in the nervous system

Changes of intracellular osmolarity under normal physiological extracellular osmotic pressure (isosmotic stress) or anisosmotic volume stress (under hypertonic /hypotonic extracellular osmolality conditions) can trigger cellular responses in vertebrate cells to regulate volume [20, 21]. In hypotonic extracellular conditions, water enters into cells and causes cell swelling, which triggers regulatory volume decrease (RVD), a homeostatic counter-response [22]. RVD results from the efflux of K^+^ and Cl^-^ via KCCs (KCC1, KCC3, and KCC4), and/or via K^+^ and Cl^-^ channels along with water. The canonical volume-regulated KCCs are for the most part inactive under isotonic conditions but activate in response to cell swelling [13, 14]. On the contrary, cell shrinkage due to hypertonic conditions elicits regulatory volume increase (RVI) [20], which results in Na^+^ and Cl^-^ influx via Na^+^ channels, activation of NKCC1, or Na^+^/H^+^ exchange. Activation of NKCC1 and/or reduction of KCC function can cause an increase in intracellular Na^+^ and Cl^-^ levels, which can result in isosmotic cell swelling [23]. Cell swelling due to either isosmotic and anisosmotic conditions could contribute to swelling in neurons, glia, and capillary endothelial cells of the blood-brain barrier (BBB), which can collectively contribute to brain edema and will be discussed further [24].

The WNKs are a serine/threonine kinase family encoded by the genes WNK1-4 [25]. In the context of volume regulation, protein phosphorylation effectuated by the WNK-SPAK kinase pathway activates NKCC1 but simultaneously inhibits KCCs, whereas protein dephosphorylation inhibits NKCC1 and activates the KCCs [26]. The major phosphoregulatory sites have been identified at the N-terminus of NKCC1 at Thr^203^, Thr^207^ and Thr^212^, and in the C-terminus of KCC1-4 at Thr^991^ in KCC3 and at Thr^906^ in KCC2 in human, sharks and mice [4, 8]. Sites homologous to KCC3 Thr^991^ and Thr^1048^ residues, Site-1 (Thr^991^) and Site-2 (Thr^1048^), are phosphorylated in all human KCCs [8]. Substitution of these Thr residues (with Ala) causes KCC2 and KCC3 activation due to inhibition of phosphorylation [27, 28]. Recently, a functional kinomics study showed the WNK3-SPAK complex to be vital for regulating phosphorylation of KCC3 at Site-1 and Site-2 [26].

**Figure 1.**
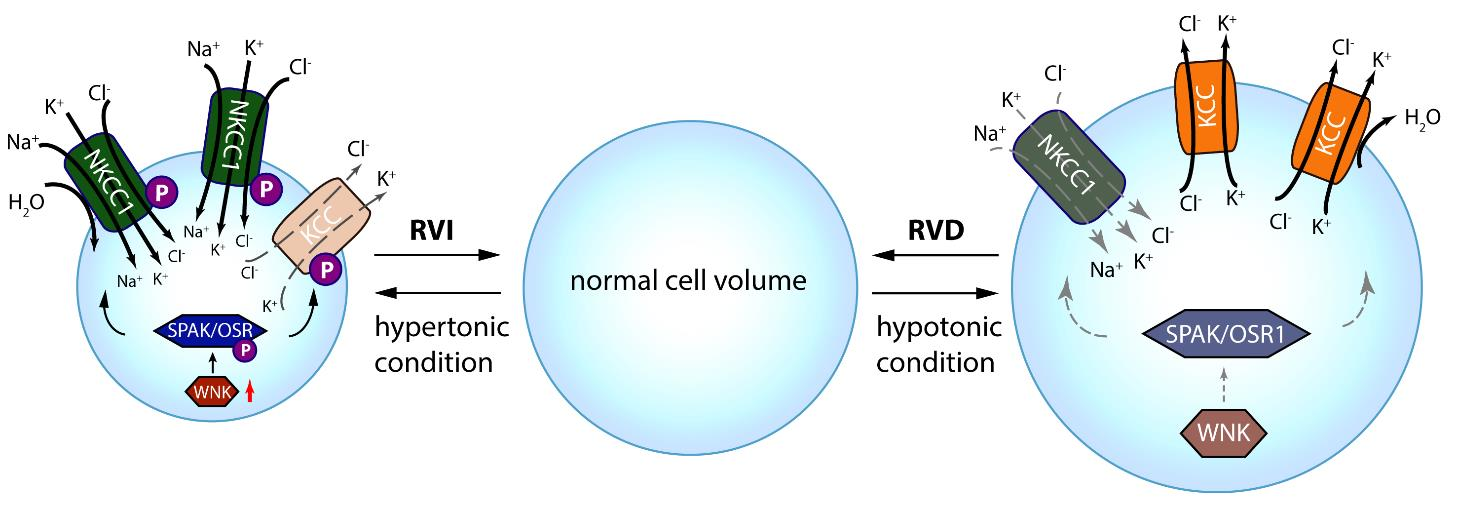
Roles of CCC in cell volume regulation Intracellular osmolarity changes trigger cellular responses for volume regulation. Under hypertonic extracellular conditions, water extrudes from the cells and causes cell shrinkage, triggering a counter-response of regulatory volume increase (RVI). In this condition, the WNK-SPAK/OSR1 pathway is activated and phosphorylates NKCC1 and KCCs, resulting in NKCC1activation and KCCs inhibition. This subsequently leads to influx of Na^+^, K^+^ and Cl^-^ via NKCC1 along with water, thus restoring cell volume. On the contrary, cell swelling due to hypotonic stress elicits regulatory volume decrease (RVD), in which the WNK-SPAK/OSR1 pathway remains inactive and NKCC1 and KCCs are dephosphorylated. This results in NKCC1 inhibition but stimulation of KCCs, which lead to KCC-mediated efflux of K^+^ and Cl^-^ along with water, and cell volume decrease.

SPAK/OSR1 interacts with KCC1-4 and NKCC1 by means of a specific conserved carboxyl-terminal (CCT) domain [26, 29, 30]. SPAK’s CCT domain is recognized by the Arg-The-Xaa-Val/Ile (RFXV/I) domain in the amino-terminal of KCC1-4 and NKCC1-2. KCC2 subtype KCC2b has no RFXV/I motif, so SPAK overexpression decreases the transport activity of KCC2a but not KCC2b [5, 31]. This ability of SPAK/OSR1 (via CCT) to attach to both the WNKs and CCCs (upstream and downstream) is essential for coordinating cellular cotransporter activity in isosmotic or hyperosmotic conditions [30, 32]. WNK’s binding to SPAK/OSR1 allows the phosphorylation of residues found in the T-loop of the SPAK catalytic domain [30, 32]. This process is crucial for SPAK’s ability to phosphorylate (inhibit) Thr^991^/ Thr^1048^ in KCC3 and Thr^906^ in KCC2 while phosphorylating (activating) NKCC1 at Thr^203^/Thr^207^/Thr^212^ in response to cellular shrinkage and hypertonicity (Fig. 1) [5]. NKCC1’s phosphorylation and activation by SPAK/OSR1 in hypertonic conditions is a vital component of RVI [5, 33], which leads to influx of Na^+^, K^+^ and Cl^-^ along with water to achieve cell volume recovery (Fig. 1). Under hypotonic extracellular conditions, water enters the cells and causes cell swelling, subsequently triggering a counter volume regulation response, i.e., RVD. In this condition, the WNK-SPAK/OSR1 pathway remains inactive and inhibits NKCC1 activity (Fig. 1). Phosphatase-mediated dephosphorylation of KCCs stimulates their activity and leads to efflux of K^+^ and Cl^-^ along with water, and thus decreases cell volume (Fig. 1). Therefore, pharmacological or genetic antagonism of WNK-SPAK/OSR1 enables cellular chloride expulsion by simultaneously decreasing phosphorylation of NKCC1 and the KCCs, and thus acute cell swelling caused by osmotic stress may be prevented or the cerebral edema caused by energy failure diseases may be mitigated [26, 34, 35].

**Table 1:** Phenotypes of transgenic knockout (KO) mice involving WNK-SPAK/OSR1-CCC complex

Protein	Co-transport ions	Tissue and Subcellular Distribution	Relating to human diseases	Phenotypes of KO/KI mice	Refs.
SLC12A1 (NKCC2)	Na^+^, K^+^, Cl^-^	Kidney-specific (TAL); apical plasma membrane; hypothalamo-neurohypophyseal system	Bartter syndrome	Severe hypotension, hypokalemia, hypercalciuria, metabolic alkalosis	[80-82]
SLC12A2 (NKCC1)	Na^+^, K^+^, Cl^-^	Ubiquitous; basolateral plasma membrane	None	Sensorineural deafness, alterations in endolymph secretion, reduced saliva production, sensory perception abnormalities and infertility	[58, 83-86]
SLC12A3 (NCC)	Na^+^, Cl^-^	Kidney-specific (DCT); bone? apical plasma membrane	Gitelman syndrome	Hypotension, hypocalciuria, hypomagnesemia, hypokalemia	[87-89]
SLC12A4 (KCC1)	K^+^, Cl^-^	Ubiquitous	None	No phenotype	[90]
SLC12A5 (KCC2)	K^+^, Cl^-^	Neuron-specific; basolateral plasma membrane (?)	Epilepsy	Complete - death due to absent respiratory drive. Incomplete KO (5%of function remains). Status epilepticus, death ± 15 days after birth.	[91, 92]
SLC12A6 (KCC3)	K^+^, Cl^-^	Widespread	Andermann syndrome	Knockout mice recapitulate the locomotion and neuropathy phenotypes and demonstrate axonal swelling	[92, 93]
SLC12A7 (KCC4)	K^+^, Cl^-^	Widespread; basolateral plasma membrane (?)	None	Sensorineural deafness and renal tubular acidosis	[94, 95]
SPAK	--	Ubiquitous	No report in human, but resemble Gitelman syndrome in mice	KO mice exhibited hypotension and recapitulated Gitelman syndrome with hypokalemia, hypomagnesemia, and hypocalciuria; higher nociceptive threshold and increased anxiety	[12, 66]
OSR1	--	Ubiquitous	No report in human, but resemble Bartter syndrome in mice	Global KO - die in utero. Heterozygous KO - low BP. Kidney tubule-specific KO - normal BP with hypercalciuria and hypokalemia	[96, 97]
WNK1	--	Ubiquitous	HSANⅡ; PHAII	Less susceptible to hypersensitivity to cold and mechanical stimuli after peripheral nerve injury	[73, 75, 96, 98, 99]
WNK2	--	Prominently expressed in brain neurons; fetal brain and heart	No report	No report	[100, 101]
WNK3	--	Ubiquitous	Autistic disorder?	Mice exhibited less cytotoxic edema after MCAO; compensated elevation of WNK1/SPAK axis in the kidney	[34, 65, 72, 102]
WNK4	--	Ubiquitous	PHAII	KO mice exhibited hypokalemia but normalcalciuria	[99, 103]

## Activation of WNK-SPAK /OSR1 kinase complex by low intracellular Cl^-^

The WNK kinases are not only effector kinases that work in conjunction with the SPAK/OSR1 kinases to regulate CCCs by phosphorylation, but may also serve as the intracellular Cl^-^ sensors (potentially cell volume sensors) [6]. Cl^-^ is a vital ion which is involved in the regulation of neuronal excitability and cell volume [20, 36]. Cl^-^ stabilizes WNK1 in its inactive conformation and prevents kinase autophosphorylation by binding to WNK1’s catalytic site, which is situated at the DLG motif and the N-terminus of the activation loop [6]. Because WNK1 is sensitive to Cl^-^-mediated inhibition of autophosphorylation, it effectively serves as a Cl^-^ concentration sensor [6]. Among WNK1-4, Cl^-^ inhibits WNK4 kinase activity at lower concentrations than it inhibits activity of WNK1 or WNK3 [37]. In light of expression of WNK4 in the BBB endothelium and increased level in ischemic stroke of hypertensive rats [16], future studies are needed to elucidate roles of WNK4 in the CNS, especially considering to its high sensitivity to [Cl]_i_. Developing inducible and cell-type specific WNK4 KO mice will be helpful to investigate these questions.

Furthermore, the functions of SPAK and/or OSR1 are also sensitive to the changes in Cl^-^ concentration [38]. In the adult central nervous system (CNS), GABA is one of the main inhibitory neurotransmitters, utilizing the activated ligand-gated, Cl^-^-permeable GABA_A_ receptors (GABA_A_Rs) to propagate its fast synaptic hyperpolarizing effect [39]. When GABA binds to and activates the GABA_A_ receptor, the Cl^-^ ions flow into the cell through the channel, resulting in hyperpolarization of the cell and suppression of the electrophysiological activity of the related neurons [39]. As opposed to NKCC1, increased KCC2 expression during brain maturation mediates Cl^-^ extrusion and reduces [Cl^-^]_i_ in neurons, which in turn favors GABA_A_R-mediated hyperpolarization in the mature CNS [40]. The low [Cl^-^]_i_ maintained by KCC2 is required for GABA-mediated synaptic inhibition [41, 42]. However, in developing neurons, responses actuated by GABA_A_Rs are depolarizing and sometimes excitatory, which are critical for synaptogenesis, neuronal proliferation, and migration [43, 44]. For example, there is a “excitatory-inhibitory developmental sequence” of GABA in the nervous system, which is closely related with the [Cl^-^]_i_ [45-52]. Increase in expression and activity of WNK3 and OSR1 was believed to be the cause of disturbance of GABA-mediated neurotransmission in CNS and consequently schizophrenia [44, 46]. Neuropathic pain and spasticity resulting from spinal cord injury (SCI) are often associated with defective GABAergic function, mainly caused by impairment of KCC2 functionality [49, 53]. All these studies signal for importance of [Cl^-^]_i_ in regulating WNK. In the peripheral nervous system (PNS), glia and neurons have a relatively higher [Cl^-^]_i_ due to KCC3’s inhibition, elevated expression of NKCC1, and an absence of KCC2 [54, 55]. In response to this higher [Cl^-^]_i_, WNK can be deactivated and lead to dephosphorylation of NKCC1 and KCC3, resulting in decreased NKCC1 expression and increased KCC3 expression and activation, which in turn reduce [Cl^-^]_i_ and favors GABA-mediated hyperpolarization. Thus, neurons and glia of the PNS are more efficient in volume regulation and GABAergic circuit functions due to KCC3-mediated K^+^ and Cl^-^ efflux and high-water permeability [56, 57]. Taken together, WNK-SPAK/ OSR1-mediated phosphoregulation of NKCC1/KCCs might be important for determining the molecular mechanisms in diseases characterized by neuronal hyperexcitability, such as stroke and epilepsy, where lack of GABA-mediated inhibition might be responsible for the excitatory effects.

**Figure 2.**
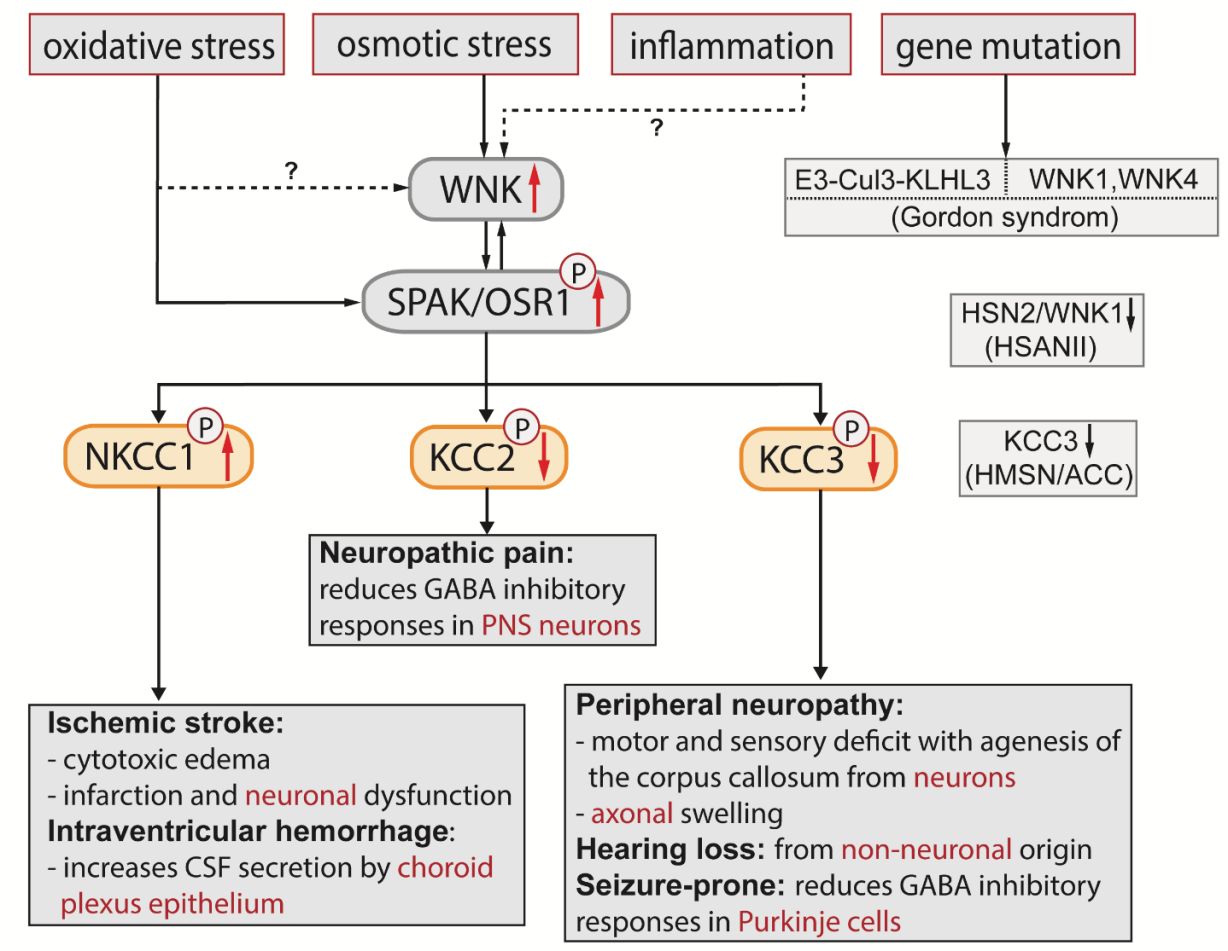
Illustration of the WNK-SPAK/OSR1-CCCs cascade in nervous and non-nervous system diseases Mutations of E3 ubiquitin ligase components cullin 3 (CUL3) and kelch-like 3 (KLHL3) were identified to cause Pseudohypoaldosteronism type II (PHAII) with increased WNK1 and WNK4 abundance in kidney. Gene mutations in WNK1 and WNK4 also cause PHAII with compromised cell volume homeostasis. In addition, osmotic stress can trigger WNK-SPAK/OSR1 complex activation, which leads to downstream phosphorylation of CCCs, especially stimulatory phosphorylation of NKCC1 and inhibitory phosphorylation of KCCs. Overstimulation of NKCC1 increases cytotoxic edema, enlarges infarction, and worsens neurobehavioral function in ischemic stroke. Hyperactive NKCC1 increases CSF secretion by the choroid plexus epithelium and causes post-hemorrhagic hydrocephalus after intraventricular hemorrhage. On the other hand, phosphorylation of KCC2 by WNK-SPAK/OSR1 decreases its Cl^-^ efflux and reduces GABA-mediated inhibition of spinal nerve transmission and causes neuropathic pain. To date, no direct evidence links oxidative stress or inflammation to WNK activation in the nervous system. However, oxidative stress can directly activate SPAK/OSR1, which in turn regulates WNK activity, thus indirectly activates WNK; inflammation-induced stimulation of the WNK-SPAK/OSR1 pathway could also increase WNK activity. Dysfunction of KCC3, such as via KCC3 mutation, leads to compromised cell volume homeostasis and causes hereditary motor and sensory neuropathy with agenesis of the corpus callosum (HMSN/ACC), hearing loss and a reduced threshold for seizure. Hereditary sensory and autonomic neuropathy type II (HSANII) caused by HSN2 gene mutations leads to a loss-of-function for WNK1 activity. Taken together, the WNK-SPAK/OSR1-CCC signaling pathway emerges as a new therapeutic target for nervous and non-nervous system disorders.

## Neurological deficits in transgenic mice with modifications of WNK-SPAK/OSR1-CCC signaling pathway

In the past decade, establishment of specific gene knockout (KO) models or genetic knock-in (KI) mouse models significantly improved our understanding of the physiological function of the WNK-SPAK/OSR1-CCC pathway in the nervous system. Global NKCC1 KO mice exhibited seizure, hearing loss (due to loss of K^-^ secretion in the inner ear and suppressed bumetanide-sensitive ^86^Rb uptake and chloride fluxes across the secretory epithelium), and slightly diminished blood pressure than wild-type (WT) animals (Table 1) [58]. KCC3 and KCC4 are copiously expressed in both CNS and PNS, and mutations of them can cause various diseases [59]. Two KCC3 KO mouse models have been created for further exploration of the functions of KCC3 [60, 61]. Using homologous recombination truncating mutations to disrupt the SLC12A6 gene of KCC3 generates KCC3 KO mice (SLC12A6^-/-^) [60]. The second KCC3 KO mouse line was created by Boettger et al [61], as well as a spontaneous mutation in the Jackson Lab [62]. KCC3 KO mice (SLC12A6^-/-^) show discoordination and feebleness in the hind limbs 14 days after birth, as well as axonal swelling in the sciatic nerves, hypomyelination, demyelination, and fiber degeneration (Table 1) [63]. In two independent studies, nerve conduction impairment in KCC3 KO mice arose from the combination of impaired ionic clearance and axonal swelling in the sciatic nerve [63, 64]. Adult KCC3 KO mice showed reduced exploration of their environment and deafness in behavior tests due to abnormal inner ear cells [60, 61]. Furthermore, in cerebellar slice preparations (which express abundant KCC3), both WT and KCC3-null Purkinje cells exhibit swelling in hypotonic conditions but KCC3-null cells failed to normalize their volume after returning to isotonic conditions, suggesting that KCC3-dependent RVD was impaired in KCC3 KO cells [61]. Additionally, the seizure threshold of KCC3-null mice is lower than WT mice, which was detected by the patch-clamp technique [61].

Inhibition of the WNK-SPAK-CCC cascade can reduce cellular swelling in ischemic stroke brains by concurrently inhibiting NKCC-mediated ionic influx and stimulating KCC-mediated ion efflux. WNK3 KO mice show normal weight, behavior, and growth [35, 65]. They exhibited less cytotoxic edema after ischemic stroke in the middle cerebral artery occlusion (MCAO) model [34]. However, both adult WNK3 KO and SPAK KO mice have impaired locomotor function (Table 1) [34]. Compared to their WT counterparts, SPAK KO mice display a higher nociceptive threshold [66]. They also exhibit increased thigmotaxis in the open field chamber and spend significantly longer periods of time in the dark area of the light/dark box, indicating an anxiety-like phenotype [66]. These findings suggest that WNK-SPAK-CCC signal pathway is important in regulating function of the nervous system, which may be through fine tuning of Cl^-^ and cell volume homeostasis.

## The Cl^-^ cotransporters and WNK-SPAK signaling pathway in ischemic brain edema and hypersecretion of cerebrospinal fluid (CSF)

The WNK-SPAK-CCC signaling complex plays a role in regulating cell volume and ionic homeostasis in both the CNS and PNS. Considering the special physiology of the brain and skull, cell volume regulation in the CNS is especially important because slight volume changes can affect normal brain function and cause life-threatening problems [21]. Such as in ischemic stroke, activation of NKCC1 causes brain edema (isosmotic cell swelling via increasing the intracellular concentration of Na^+^ and Cl^-^) and brain injury [16, 67]. Brain edema can increase intracranial pressure, trigger herniation, or even cause death. In a recent experimental ischemic stroke study, WNK3 KO mice displayed significantly decreased cerebral swelling, axonal demyelination, and infarct volume as well as accelerated neurobehavioral recovery, compared to WT mice [35]. WNK3 KO mice have shown two swelling-associated components of ischemic cerebral edema to be mitigated: endothelial and perivascular cytotoxic edema of astrocytes [26]. These effects likely result from decreased NKCC1 activity and increased KCC3-mediated cellular Cl^-^ efflux [26].

The neuroprotective phenotypes conferred by WNK3 KO were associated with a decrease in NKCC1 cell surface expression, reduced phosphorylation at NKCC1 stimulatory sites (Thr^203^/Thr^207^/Thr^212^) and a decrease in stimulatory hyperphosphorylations of the SPAK/OSR1 catalytic T-loop [35]. Compared to WT mice, WNK3 KO and SPAK KO (or SPAK heterozygous) mice showed more than a 50% reduction in infarct volume and associated cerebral edema after ischemic stroke. Cultured primary neurons and oligodendrocytes showed increased tolerance to *in vitro* ischemia with genetic inhibition of WNK3 or small interfering RNA knockdown of SPAK/OSR1 [34]. Therefore, WNK-SPAK-NKCC1 complex inhibition has therapeutic potential for treating cerebral edema and ischemic brain injury.

In addition, a new report shows that the activation of SPAK, which stimulates NKCC1 at the choroid plexus epithelium (CPE) apical membrane, mediates the intra-ventricular hemorrhage (IVH)-induced hypersecretion of CSF in a rat model of post-hemorrhagic hydrocephalus (PHH) [68]. Genetic depletion of SPAK normalizes hyperactive CSF secretion rates and reduces PHH symptoms [68]. These findings suggest that pharmacological targeting WNK-SPAK-CCCs signaling pathway presents a novel strategy to treat patients with hydrocephalus instead of invasive CSF shunting.

## The Cl^-^ cotransporters and WNK-SPAK signaling pathway in other nervous system diseases

In humans, mutations of KCC3 can cause Andermann syndrome, a Mendelian disease characterized by agenesis of the corpus callosum with peripheral neuropathy (ACCPN) [60]. T991A mutations of KCC3 in patient cells stop Thr (991) phosphorylation and cause constitutive KCC3 activity, which interferes with cell volume regulation [55]. Mice with KCC3^T991A/T991A^ mutations displayed constitutive KCC3 activity and recapitulated aspects of the electrophysiological, histopathological, and clinical findings of peripheral neuropathy patients [55]. On the other hand, the “loss of function mutations” in KCC3 transporters, mostly due to premature termination, cause hereditary motor and sensory neuropathy (HMSN) with agenesis of the corpus callosum (ACC) [69]. The disorder is characterized by an early onset sensorimotor neuropathy associated with variable degrees of ACC. Individuals are affected at the rate of 1:2117 live births (in the regions of Charlevoix and Saguenay-Lac-Saint-Jean) and carry two copies of a mutant allele which causes KCC3 truncation (Table 1) and loss of transport activity [60]. There are two kinds of gene mutations of T991A and pT813X. KCC3 KO mouse models recapitulate the neuropathy phenotype of the patients [60, 61]. KCCs also play an important role in the maintenance of nervous system function. Epilepsy is a common CNS disease, and the patients with “loss of function mutations” of KCC3 exhibit a lower seizure threshold, which was also observed in KCC3 KO mice [61]. The complex pathophysiology phenotypes in the nervous system resulting from loss-of-function or gain-of-function of KCC3 present challenges for developing therapeutic treatment by targeting KCC3. However, it is still unclear how KCC3 is involved in these diseases. Moreover, the time point when KCC3 function is critical during development or lifetime is still unclear. Therapeutic approaches specifically targeting KCC3 can be difficult due to a list of unknown cell types that could be potentially affected by KCC3 disruption [70, 71]. Therapies targeting KCC3 is also likely to affect the other KCC isoforms due to the high level of conservation in KCC3 among all isoforms [55, 71]. Improved gene therapy is needed for more specific targeting at KCC3 without disrupting the other isoforms.

Additional reports show that WNK gene families are involved in other neurological diseases. A small number of patients with autism revealed deletions of the WNK3 and FAM120C genes, for which a definitive phenotype has not been previously characterized [72]. Additionally, a genetic disease known as hereditary sensory neuropathy (HSN) or hereditary sensory and autonomic neuropathy (HSAN) was initially believed to be caused by a mutation in a single exon open reading frame (ORF) located within intron 8 of the WNK1 allele, termed “HSN2” [73]. HSN2 was later found to be a nervous system-specific alternately spliced exon of WNK1 as early as 2008 [74]. HSANII affects the peripheral and spinal nerves and results in loss of touch, temperature, and pain sensation [75]. WNK1/HSN2 contributed to a maladaptive decrease in the activity of the KCC2 by increasing its inhibitory phosphorylation at Thr^906^/ Thr^1007^, resulting in an associated loss of GABA-mediated inhibition of chronic pain hypersensitivity after nerve injury [75]. It was found that both genetic knockout and pharmacologic inhibition of WNK/HSN2 leads to amelioration of neuropathic pain while not producing neurological deficits [75]. These findings reveal therapeutic potentials of targeting WNK1 and KCC2 for neuropathic pain after nerve injury [75]. However, KCC2 KO mice died immediately after birth due to severe motor deficits that prevented respiration [76].

## Conclusions

For decades, cell volume regulation has been a subject undergoing intense study. The WNK-SPAK/OSR1 kinase complex and Cl^-^ cotransporters play a crucial role in regulating cell volume in the nervous system (Fig. 2). Identification of specific human gene mutations in the WNK-SPAK-CCC signaling pathways and development of genetic mouse models enhanced our understanding of the significance of cell volume homeostasis in nervous system disease development and treatment. Oxidative stress directly activates SPAK/OSR1, which can in turn regulates WNK activity, thus indirectly activates WNK (Fig. 2) [30]. Injuries such as peripheral nerve injury, spinal cord injury, and ischemic stroke are also shown to trigger WNK activation (Fig. 2). However, no evidence yet directly links inflammation to WNK activation. Gene mutations in WNK1 and WNK4 lead to compromised cell volume homeostasis and cause PHAII [77], the phenotypic inverse of the salt-wasting Gitelman syndrome (Fig. 2). Other genetic mutations in the ubiquitin E3 ligase components cullin 3 (CUL3) and kelch-like 3 (KLHL3), the upstream genes encoding negative regulators of WNK, increases WNK abundance [77-79], HSN2 gene mutations lead to a loss-of-function on WNK1 activity and lower the pain threshold through KCC2-mediated GABA hyperpolarization [75]. Therefore, blocking WNK-SPAK/OSR1 may provide a potent means of synchronously inhibiting of NKCC1-mediated Cl^-^ influx and enhancing KCC-mediated Cl^-^ extrusion for multiple syndromes exhibiting depolarized GABA responses and altered Cl^-^ homeostasis, cerebral edema, or hydrocephalus. Mutations of WNK-SPAK-CCC complex identify them as therapeutic targets for other nervous system or non-nervous system disorders.
